# Pre-incisional infiltration of tonsils with dexamethasone dose not reduce posttonsillectomy vomiting and pain in children

**DOI:** 10.4103/1658-354X.57874

**Published:** 2009

**Authors:** Kamran Montazeri, Ahmad Okhovat, Azim Honarmand, Mohammad Reza Safavi, Leila Ashrafy

**Affiliations:** *Department of Anesthesiology and Intensive Care Medicine, Isfahan, Iran*; 1 *Department of Otolaryngology, Isfahan University of Medical Sciences, Isfahan, Iran*

**Keywords:** *Dexamethasone*, *postoperative pain*, *tonsillectomy*, *vomiting*

## Abstract

**Background and Objective:**

Recently, dexamethasone has been found to have a prophylactic effect on postoperative vomiting and pain in children undergoing tonsillectomy. However, few studies have examined the preemptive analgesic effects of dexamethasone after tonsillectomy. The aim of this study was to evaluate the effect of pre-incisional infiltration of tonsils with dexamethasone on the incidence and severity of postoperative pain and vomiting in children undergoing tonsillectomy under general anesthesia.

**Materials and Methods:**

In a double blinded study, 62 patients were randomly allocated to infiltrate dexamethasone (0.5 mg/kg, maximum dose, 12 mg) or an equivalent volume of saline at the peritonsillar region. All infiltrations were performed following the induction of general anesthesia and 5 minutes prior to the onset of surgery. Anesthetic agents, end-tidal carbon dioxide levels, and the administration of intravenous fluids were carefully regulated. Surgery was performed by one attending otolaryngologists using the same dissection and snare technique. The incidence of pain and vomiting, need for rescue antiemetics, and analgesic consumption were compared in both groups. Pain scores used included Children's Hospital Eastern Ontario Pain Scale, “faces”, and a 0-10 visual analogue pain scale.

**Results:**

Demographics of dexamethasone and placebo groups were similar. No statistically significant difference was found between the dexamethasone and placebo groups in pain score, nausea, vomiting, irritability, or analgesic requirement postoperatively.

**Conclusion:**

Preincisional infiltration of the tonsils with dexamethasone play a limited role in the recovery phase from tonsillectomy, but further prospective, randomized studies are needed to support it.

## INTRODUCTION

Tonsillectomy is one of the most frequently performed ambulatory surgical procedures in children and is associated with an incidence of postoperative vomiting ranging between 40% and 73%.[1] The introduction of an electrodissection surgical technique has virtually eliminated immediate postoperative hemorrhage. However, it may cause more pain, discomfort and poor oral intake due to more local inflammation, nerve irritation and laryngeal muscle spasm.[2] Postoperative pain is a significant problem that we believe continues to be undertreated in the pediatric population. As this procedure has moved from the inpatient to the outpatient arena, adequate control of postoperative pain has proved to be a difficult and complex task. Preemptive analgesia (blockade of pain receptors) has been shown to ameliorate postoperative pain after a number of surgical procedures.[3–5] The effectiveness of preemptive anesthesia was shown in a recent study of tonsillectomy patients treated with preincisional ropivacaine and clonidine.[6] Although narcotic analgesics are the mainstay of pain control, they do not completely control pain and are often discontinued secondary to side effects, particularly nausea and vomiting. Another intervention is obviously required to assist the early phase of tonsillectomy recovery. Dexamethasone, given intravenously as a single intraoperative dose, has been studied extensively in the recovery of patients after tonsillectomy. Improvement in nausea, diet, trismus, and pain has been reported,[7,8] although not all authors have found such significant effects.[9,10] A meta-analysis of randomized studies showed less nausea and vomiting and improvement in the resumption of soft diet at 24 hours.[11] Because of its potential to lessen the morbidity that children experience in the first 2 days after surgery, we embarked on this study to examine the effects of pre-incisional infiltration of dexamethasone on vomiting and pain in children undergoing tonsillectomy.

## MATERIALS AND METHODS

A randomized, double-blind, placebo-controlled clinical trial was conducted at the Alzahra Hospital following approval by the Hospital Ethics Committee. Informed written consent was obtained from the parents of 62 ASA I and II children aged between three and 15 years scheduled for elective inpatient tonsillectomy with or without adenoidectomy. Exclusion criteria included a known allergy to dexamethasone, any patient with a known contraindication to steroid use, an ASA physical status III or greater, a history of severe postoperative vomiting, children who received antiemetics, steroids, anti-histaminic, or psychoactive drugs during the week before surgery, patients who need additional analgesic during surgery, and children with sever postoperative bleeding who were reoperated. In both groups, no oral premedication was administered before surgery. After IV infusion of acetated Ringer's solution 10 mL/kg, anesthesia was induced with fentanyl 1-2 μg/kg and thiopental 5 mg/ kg IV, and the trachea was intubated after the administration of atracurium 0.5 mg/kg IV. Anesthesia was maintained with halothane 1.0% and N_2_ O in 50% oxygen under mechanical ventilation adjusted to maintain heart rate and blood pressure values within 20% of the baseline induction. Muscle paralysis was maintained with atracurium. The patients were given intravenous morphine 0.1 mg/kg for intraoperative analgesia. During anesthesia, the heart rate (ECG), arterial oxygen saturation (SpO_2_), blood pressure, temperature and end-tidal CO_2_ were monitored. Following the induction of general anesthesia, but prior to the onset of surgery, the peritonsillar regions were infiltrated. The same surgeon, using an electrodissection technique, performed all operations. The patients were randomized to infiltrate dexamethasone (0.5 mg/kg, maximum dose 12 mg) or an equivalent volume of saline at peritonsillar region. Study drugs were marked only with a coded number label. A computer-generated table of numbers guided randomization. All infiltration was performed in the same manner. The needle was inserted superficially into the tonsillar pillar, and following aspiration the pillar was ballooned. Three injections into both pillars were made on every patient: At superior pole, at the inferior pole, and between the poles. A reversal dose of neostigmine 40 μg/kg with atropine 20 μg/kg was administered at the completion of surgery. At the end of surgery, gastric contents were suctioned via an orogastric tube before extubation. The trachea was extubated when the child was awake. All children were transferred to the (PACU). Each patient was assessed by a single-blinded observer measuring postoperative pain in the recovery room prior transfer to the ward and then again at four and eight hours. In patients aged 12 years and older, we used a simple linear analog pain scale from 0 = no pain to 10 = most severe.[12] The Oucher Faces Scale was used in patients aged 3 to 11 years.[13] This consists of a series of six photographs depicting a child in various degrees of distress corresponding to a numeric scale of 0 to 10. Each child's comprehension and proficiency at using this scale was examined at the preadmission clinic. No child was excluded from this study because of an inability to use the Oucher Faces Scale. The FLACC scale was also used for scoring of irritability at postanesthesia care unit (PACU) after tonsillectomy.[14] Each patient rated his or her pain, with the help of the nurse, at 24, and 8 hours following surgery. The time and dosage of postoperative analgesics received was also recorded. For a pain score of 3 or greater, i.v. meperidine (Pethidin, Alodan^®^, Gerot, Iran) was administered in increments of 5 mg (initial dose for children <20 kg, 5 mg, 20 kg, 10 mg) every 5 min until the child was comfortable. Water was offered to children one hour after arrival in the PACU and at the child's request. Intravenous fluid infusion was continued until adequate oral intake (oral ingestion of 100 mL of fluids and 100 mL of soft food within four hours). The nurse recorded the incidence of vomiting. For the purpose of this study, vomiting was defined as ‘the forceful expulsion of liquid or solid gastric contents’. Retching and nausea were not considered vomiting. Repeated vomiting within a one-to two-minute period was recorded as a single emesis. Vomiting occurring more than twice was treated with metoclopramide 0.15 mg·kg–1 i.v. The presence or absence of any side effect such as bleeding, fever, flushing or headache was noted.

### Statistical methods

A sample size calculation performed before the commencement of the study revealed that 31 patients per group would be required to detect a 30% reduction in the incidence of postoperative pain, assuming a 40% baseline incidence of pain after tonsillectomy in the control group (α ≤ 0.05 and β = 0.20). Data were analysed using the statistical program SPSS for window, Release 11. Parametric data including patient demographics of age and weight were analyzed using Student's *t* test or Chi-squared test for contingency tables as appropriate. Visual analogue scores were analyzed using Mann-Whitney U test. Differences within groups were subjected to the Wilcoxon signed rank test. A *P* value < 0.05 was considered statistically significant.

## RESULTS

**Figure 1:**
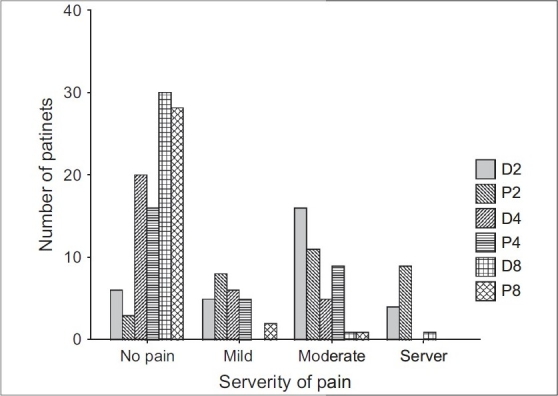
Incidence and severity of pain at 2, 4, and 8 hours after tonsillectomy in dexamethasone (D) or placebo (P) group. There were no significant differences in severity of pain between the two groups

The two groups did not differ significantly with respect to age, sex, weight, length of operation, and fluid required during the operation [Table 1]. The incidence and severity of postoperative pain at 2, 4, and 8 hours after tonsillectomy were not statistically significantly different between the two groups [Figure 1]. The incidence of nausea and vomiting at 2, 4, and 8 hours after tonsillectomy were not significantly different between the two groups [Table 2]. There was no significant difference between the two groups in the incidence of irritability at PACU after tonsillectomy [Table 3].

**Table 1:** Patient demographic and clinical data

Parameter	Placebo	Dexamethasone
No	31	31
Age (yr)	8.8 ± 5.00	9.6 ± 13.00
Weight (kg)	25.5 ± 13.8	25.6 ± 13.50
Gender, n (%)		
Male	14 (45.2)	15 (48.4)
Female	17 (54.8)	16 (51.7)
Procedure length (min)	31.4 (6.1)	32.6 (7.3)
Fluid requirement (mL/kg)	17.1 (4.3)	15.3 (4.1)

No statistical difference was detected between the two groups (*P* > 0.05). Values are expressed as mean ± SD

**Table 2:** Incidence of nausea and vomiting at 2, 4, 8 hours after tonsillectomy

Hours after tonsillectomy	Nausea and vomiting	Number of patients in placebo group	Number of patients in dexamethasone group	Total number of patients
2	Yes	7	5	12
	No	24	26	50
	Total number of patients	21	31	62
4	Yes	5	4	9
	No	26	27	53
	Total number of patients	31	31	62
8	Yes	0	0	0
	No	31	31	62
	Total number of patients	31	31	62

No statistical difference was detected between the two groups (*P* > 0.05)

**Table 3:** Incidence of irritability at postanesthetic care unit after tonsillectomy

Irritability score	Number of patients in placebo group	Number of patients in dexamethasone group	Total number of patients
0	2	10	12
1	14	9	23
2	5	4	9
3	4	5	9
4	6	3	9
Total number of patients	31	31	62

PACU - Postanesthetic care unit. No statistical difference was detected between the two groups (*P* > 0.05)

## DISCUSSION

Research into the physiology of pain has delineated 2 distinct pain mechanisms that result from the stimulus of surgical trauma. There is inflammatory pain, a local effect produced by the surgical trauma, and there is physiologic or functional pain, a central effect produced by stimulation of the central nervous system. It follows that the use of anti-inflammatory agents, such as acetaminophen, would successfully treat pain and, in fact, is standard practice in the treatment of postsurgical pain. Likewise many surgeons use “preemptive analgesia,” the use of local anesthetics at a surgical site before a surgical procedure is commenced, because this blocks the development of hypersensitivity and hyperalgesia, which are important mechanisms in the promotion central sensitization (physiologic pain).[15,16] The use of steroids, primarily dexamethasone, to decrease postsurgical pain and nausea symptoms after tonsillectomy is popular today. Systemic steroids have powerful anti-inflammatory effects and might be expected to improve recovery after surgical trauma. Several well-controlled studies have shown that dexamethasone can decrease nausea and vomiting postoperatively.[7,17] Another author found a decrease in postoperative pain.[7] In addition, less trismus,[7,8] fewer hospital admissions,[8] and quicker return to a more normal diet[18] have all been reported. Not all clinical studies, however, have shown such profound effects. Failure of dexamethasone to decrease pain was reported by several authors, although some of these studies did find other positive effects.[17,9,10,18] Decreased nausea and vomiting have not been a universal finding either.[10,18] A previous study was the foundation for this pursuit.[6] In the present study there was a trend showing a reduction in pain scores in patients who received a course of pre-incisional dexamethasone. This, however, was not found to be statistically significant. In addition, these patients required less analgesia and had fewer episodes of nausea and vomiting. This effect has been previously reported.[17,7] Dexamethasone may exert an antiemetic action via prostaglandin antagonism[19] or serotonin inhibition in the gut,[20] and release of endorphins.[21] Pain is a complex symptom, and therefore it is difficult to measure accurately.[22] Its terms of measurement and standardization depend on personal experience, social and ethnic factors, perceptual abilities, anxiety level, and the ability to describe the type and degree of pain based on some frame of reference.[23] Large numbers of subjects may be needed to prove a statistical effect. Arguably, the evaluation of pain in children is even more difficult. We rely on adjunctive indices which give us an approximation of the level of discomfort experienced. Studies are needed to develop easily reproducible and well-validated pain assessment scales.

## CONCLUSION

This study has shown that a course of dexamethasone injection before incision of tonsils may offer limited improvement in the recovery phase from tonsillectomy. It is possible that a beneficial effect was masked by insufficient sample sizes. We believe additional research is needed to find additional treatments to improve the recovery for these children.
